# Mining Electronic Health Records for Drugs Associated With 28-day Mortality in COVID-19: Pharmacopoeia-wide Association Study (PharmWAS)

**DOI:** 10.2196/35190

**Published:** 2022-03-30

**Authors:** Ivan Lerner, Arnaud Serret-Larmande, Bastien Rance, Nicolas Garcelon, Anita Burgun, Laurent Chouchana, Antoine Neuraz

**Affiliations:** 1 Inserm Centre de Recherche des Cordeliers Sorbonne Université Paris France; 2 Informatique biomédicale Hôpital Necker-Enfants Malades Assistance Publique - Hôpitaux de Paris Paris France; 3 HeKA Team Inria Paris France; 4 Inserm UMR 1163, Data Science Platform Université de Paris Imagine Institute Paris France; 5 Centre Régional de Pharmacovigilance Service de Pharmacologie Hôpital Cochin, Assistance Publique - Hôpitaux de Paris, Centre - Université de Paris Paris France

**Keywords:** COVID-19, drug repurposing, wide association studies, clinical data, pharmacopeia, electronic medical records, health data, mortality rate, hospitalization, patient data

## Abstract

**Background:**

Patients hospitalized for a given condition may be receiving other treatments for other contemporary conditions or comorbidities. The use of such observational clinical data for pharmacological hypothesis generation is appealing in the context of an emerging disease but particularly challenging due to the presence of drug indication bias.

**Objective:**

With this study, our main objective was the development and validation of a fully data-driven pipeline that would address this challenge. Our secondary objective was to generate pharmacological hypotheses in patients with COVID-19 and demonstrate the clinical relevance of the pipeline.

**Methods:**

We developed a pharmacopeia-wide association study (PharmWAS) pipeline inspired from the PheWAS methodology, which systematically screens for associations between the whole pharmacopeia and a clinical phenotype. First, a fully data-driven procedure based on adaptive least absolute shrinkage and selection operator (LASSO) determined drug-specific adjustment sets. Second, we computed several measures of association, including robust methods based on propensity scores (PSs) to control indication bias. Finally, we applied the Benjamini and Hochberg procedure of the false discovery rate (FDR). We applied this method in a multicenter retrospective cohort study using electronic medical records from 16 university hospitals of the Greater Paris area. We included all adult patients between 18 and 95 years old hospitalized in conventional wards for COVID-19 between February 1, 2020, and June 15, 2021. We investigated the association between drug prescription within 48 hours from admission and 28-day mortality. We validated our data-driven pipeline against a knowledge-based pipeline on 3 treatments of reference, for which experts agreed on the expected association with mortality. We then demonstrated its clinical relevance by screening all drugs prescribed in more than 100 patients to generate pharmacological hypotheses.

**Results:**

A total of 5783 patients were included in the analysis. The median age at admission was 69.2 (IQR 56.7-81.1) years, and 3390 (58.62%) of the patients were male. The performance of our automated pipeline was comparable or better for controlling bias than the knowledge-based adjustment set for 3 reference drugs: dexamethasone, phloroglucinol, and paracetamol. After correction for multiple testing, 4 drugs were associated with increased in-hospital mortality. Among these, diazepam and tramadol were the only ones not discarded by automated diagnostics, with adjusted odds ratios of 2.51 (95% CI 1.52-4.16, *Q*=.01) and 1.94 (95% CI 1.32-2.85, *Q*=.02), respectively.

**Conclusions:**

Our innovative approach proved useful in generating pharmacological hypotheses in an outbreak setting, without requiring a priori knowledge of the disease. Our systematic analysis of early prescribed treatments from patients hospitalized for COVID-19 showed that diazepam and tramadol are associated with increased 28-day mortality. Whether these drugs could worsen COVID-19 needs to be further assessed.

## Introduction

COVID-19 has been a global threat for public health since its emergence in China in December 2019. On July 1, 2021, more than 182 million cases of COVID-19 were reported worldwide, including more than 3.9 million deaths [1].

Multiple scientific questions have emerged over the course of the pandemic. Tremendous efforts toward finding adequate treatment options have been taken to the point that as of August 18, 2021, 2658 clinical trials were listed by the French Cochrane Centre [2]. To date, the most notable finding was that in the inflammatory phase of the disease, dexamethasone, a systemic glucocorticoid, showed a reduction in 28-day mortality among critical patients receiving respiratory support [3]. In addition, questions regularly arise regarding the safety profiles of known drugs (eg, nonsteroidal anti-inflammatory drugs [NSAIDs], angiotensin-converting enzyme [ACE] inhibitors) [4-6] or potential drug repurposing (eg, ivermectin, fluvoxamine) [7,8]. These clinical trials are motivated by in vitro efficacy of molecules [8,9], by epidemiological observations, or by both [7,10]. Furthermore, the understanding of COVID-19’s physiopathology has rapidly evolved. Hence, having understood the inflammatory component of severe cases and proven the benefit of dexamethasone in patients with severe COVID-19, dozens of immunosuppressant molecules are being tested in clinical trials [2]. At the same time, high rates of venous thromboembolism in hospitalized patients have been reported, 14.1% (95% CI 11.6-16.9) compared to 2.8%-5.6% before the pandemic [11-13], which led to multiple investigations on anticoagulant treatments.

However, 2 questions can be raised in the context of an emergent disease: (1) Are there pharmacological hypotheses that were not explored due to an incomplete physiological understanding of the disease, and (2) how can we better prioritize hypotheses to improve clinical research efficiency?

This context motivated the development of a systematic and data-driven approach that could guide clinical and epidemiological research by mining routinely collected data from electronic health records (EHRs) without the necessity of a priori knowledge. For that purpose, we took inspiration from the phenome-wide association study (PheWAS) model [14-17] to derive its drug counterpart, the pharmacopeia-wide association study (PharmWAS). This methodology analyzes in a hypothesis-free approach the association of the whole set of drug exposure with the phenotypes of a given population, similarly to a PheWAS, which analyzes the association of the whole set of phenotypes with genetic variants. The idea of PharmWAS has gained popularity in recent years under different names and has been implemented under different designs [17-20]. The PharmWAS methodology was first described by Ryan et al [17] in 2013 using a self-controlled case approach to detect adverse events. A methodology based on Cox survival models was applied by Patel et al [18] to discover drugs associated with cancer risk.

The principal challenge of a PharmWAS is to control the treatment-specific indication bias for multiple treatments. For that purpose, we developed a 2-step pipeline motivated by the literature on *causal variable selection* [21-23] that we empirically validated using reference drugs. This pipeline had to be fully data driven in order to scale to a large number of drugs. Our implementation combined a multivariate regression adjustment model and 2 PS-based methods: PS weighting and matching [24-27]. Each method represented different trade-offs between precision of the estimation and robustness to confounding. The rationale was not to report exact treatment effects, which would require domain expert knowledge supporting strong assumptions for a large set of drugs, and necessitate the strict respect of causal inference assumptions: no unmeasured confounders (exchangeability), every patient having a nonzero probability of being treated or not (positivity), and well specified models [26]. Instead, our goal was to generate pharmacological hypotheses, and we assumed that the combination of these models would reduce false-positive findings caused by indication bias.

Our main objective was to develop and validate a fully data-driven pipeline addressing these challenges. Our secondary objective was to generate pharmacological hypotheses, whether to highlight potential candidates for COVID-19 treatment or prevention or to highlight drugs worsening the condition of patients with COVID-19. To that end, we screened for associations between all drugs prescribed in the first 48 hours after admission and 28-day mortality in adults hospitalized for COVID-19 in a conventional ward.

## Methods

### Study Design and Data Sources

We performed a multicentric retrospective study using the Entrepôt de Données de Santé (EDS)-COVID database, developed upon the Assistance Publique - Hôpitaux de Paris (AP-HP) clinical data warehouse (CDW), regrouping data from 39 different sites in the Greater Paris area [28]. We used 4 types of data in this study: (1) medicoadministrative data, including diagnosis codes recorded using the *International Classification of Diseases, 10th edition* (ICD-10); (2) laboratory results from admission; (3) physiological measurements (eg, blood pressure) at admission; and (4) all medical text reports associated with inpatient stays.

### Population

**Figure 1:**
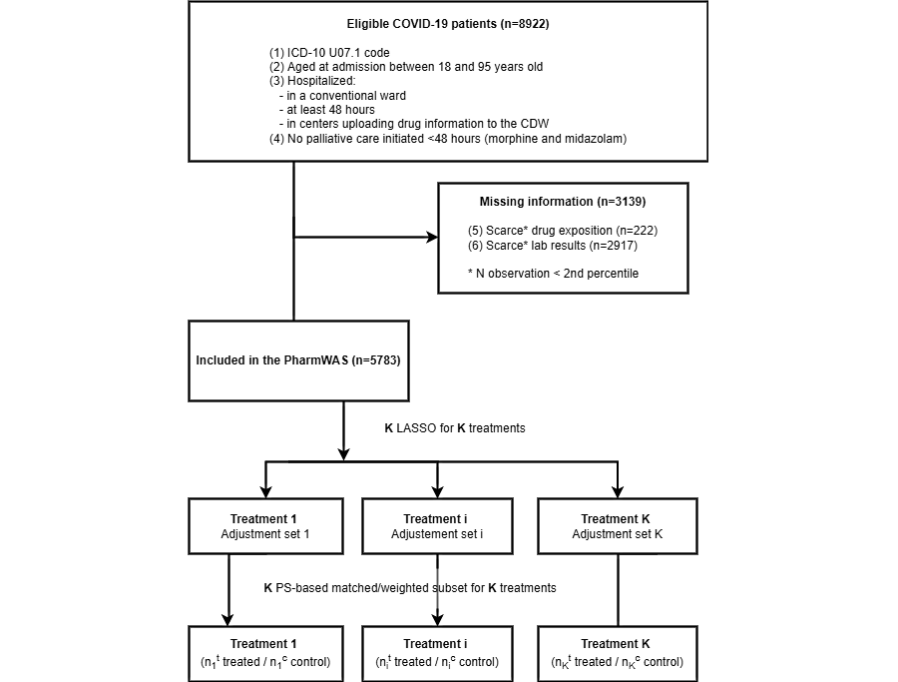
Flowchart and the PharmWAS pipeline. CDW: clinical data warehouse; ICD-10: International Classification of Diseases, 10th edition; LASSO: least absolute shrinkage and selection operator; PharmWAS: pharmacopeia-wide association study; PS: propensity score.

Selection of the study population was performed according to the following criteria: (1) first admission with an ICD-10 code of U07.1 (COVID-19), (2) age at admission between 18 and 95 years, (3) hospitalization in a conventional ward for at least 48 hours, (4) hospitalization in an AP-HP site uploading drug information to the CDW, and (5) exclusion of patients who initiated palliative therapy within 48 hours (Figure 1). The study time frame spanned from February 1, 2020, to June 15, 2021.

### Drug Exposure and Clinical Endpoint Definition

We extracted each patient's drug exposure status from the CDW corresponding to the first 48 hours after the patient was admitted for COVID-19. A code from the anatomical therapeutic chemical (ATC) classification [29] was assigned to patients with at least 1 corresponding drug regardless of the dose. We restricted the analysis to ATC level 5 codes that were present for a minimum of 100 patients. In the following sections, we use the term *drugs* to refer to these codes. The outcome was defined as all-cause 28-day mortality, with patients discharged alive before 28 days assumed to be alive at 28 days.

### Adjustment Covariate Definition

First, we included the ICD-10 codes from the current inpatient stay, restricted to chronic diseases. These codes were then grouped into broader categories using the first 3 characters and the first 2 characters of the ICD-10 system [16]. Second, we considered all laboratory results and physiological measurements. For covariates measured in at least 10% of patients, we kept only the first observation within 48 hours after admission. For covariates measured in at least 5% of patients, we kept indicator variables of the measure (1 if measured, 0 if not measured). The BMI was extracted from clinical reports using regular expressions. Finally, we added some feature engineered variables, accounting for the study period by using quintiles of the time since study initiation and quintiles of the number of measurements by source (eg, number of lab results). All continuous variables were winsorized at 2nd and 98th percentiles to account for outliers.

### Pharmacopeia-wide Association Study Pipeline

The core principle is to test for the association between each drug exposure and the outcome, controlling for covariates, given an adjustment set. This analysis is repeated n times per outcome, with n being the number of drug exposures. The results of the n tests (*P* values) obtained from this process are subsequently corrected to consider the multiple testing.

In the first step of the pipeline, we determined adjustment sets for every drug exposure, given the set of all possible pretreatment covariates. Using an adaptive LASSO procedure [30], we kept covariates associated with each drug from the subset of covariates associated with the outcome, after cross-validation of the model’s deviance. We included for each continuous covariate 3 possible forms: square root transformation, log transformation, and discretization in quintiles.

In the second step, we computed the conditional odds ratio (OR) between the drug and the outcome in a multivariate logistic regression model, given the selected covariates. In addition, we produced 2 supplementary measures of association based on PSs as secondary analyses, namely the marginal OR on the matched population and the marginal OR on the population after inverse probability weighting (IPW), restricted to the “empirical equipoise region” (EER, ie, after trimming) [27]. The EER is defined to approximate the region of *clinical equipoise*, among which uncertainty among treatment options is strong enough, so that prescribers’ preference drives the prescription instead of only patients’ characteristics [27]. PS models were fitted using multivariate logistic regression. The matching procedure was implemented with a case-control ratio of 1:4 and a caliper of 0.2 SD of the logit of the PS [31]. With IPW, the cohort was resampled by weighting each individual “i” with a weight that was based on its estimated stabilized PS π_i_ (preference score) [27]. Stabilized PS or preference scores were PS-corrected for prevalence (logit of the preference score = logit of the PS –logit of drug prevalence). Treated individuals were then weighted by 1/π_i_, and controls were weighted by 1/(1 – π_i_). Patients with stabilized PS outside the EER (ie, the stabilized PS interval of 0.3-0.7) were discarded [27]. Finally, both PS-based methods allowed the generation of automated diagnostics to assess the validity of the estimates: first, the residual imbalance in covariates, which we reported as the fraction of balanced covariates (FBC; ie, covariates with absolute standardized mean difference [ASMD] between treatment groups<0.1) [32], and second, the fraction of exposed population (FEP) remaining after applying the caliper in the matched subset or within the EER in the trimmed subset. Alpha risk inflation caused by multiple testing was addressed following the Benjamini and Hochberg procedure of the FDR (*Q*=.05), and *P* values for the OR were corrected accordingly [33].

### Validation With Treatments of Reference

We compared the data-driven determination of adjustment sets with a knowledge-based approach on 3 treatments of reference: first, dexamethasone, for which we expected a beneficial effect on 28-day mortality and which we assumed is subject to strong indication bias, and second and third, drugs of reference with an expected null effect, with high prevalence (paracetamol) and low prevalence (phloroglucinol). We studied the association of these treatments of reference with 28-day mortality on the overall population and in age-based subgroups (patients <70 or ≥70 years old). Indeed, age is the most important prognosis factor in COVID-19, and dexamethasone’s beneficial effect is heterogeneous across age subgroups [3].

We compared the association after adjusting on the data-driven adjustment set. For the knowledge-based approach, we used a set of known prognosis factors extracted from Medline articles, including age, gender, number of comorbidities, platelet count, prothrombin ratio, creatinine, blood urea nitrogen, C-reactive protein (CRP), mean arterial pressure, systolic arterial pressure, and peripheral capillary oxygen saturation [34-37].

### Missing Data Management Strategy

Missing data management followed a 2-step strategy targeting 2 different missing data mechanisms. In the first step, we excluded patients with a number of observations lower than the 2nd percentile for drugs, laboratory tests, or physiological measurements. A comparison of baseline characteristics between patients included in the analysis and patients excluded for missing data was performed to detect a possible selection bias. In the second step, we performed multiple imputation with chained equations (MICE) [38]. MICE was performed using 5 imputed data sets, and the predictive mean matching strategy was chosen, using all adjustment covariates (ie, not including drugs). The adjustment set selection was adapted to the setting of multiple imputed data sets by selecting variables that appeared in at least half of the imputed data sets. ORs were pooled according to the Rubin rule after log transformation.

In addition to these missing data–handling strategies, we also reported a measure of data missingness specific to each model, the fraction of missing information (FMI), which is considered moderately large above 0.3 and high above 0.5 [39].

### Implementation

Analyses were performed using R statistical software version 3.5.1 (R Core Team) [40]. The following packages were combined in custom functions to provide a reproducible and configurable pipeline: MICE [41], glmnet [42], MatchIt [43], and PSWeight [44]. The code is available online for transparency [45].

### Ethics

This study was approved by the Institutional Review Board of the AP-HP CDW (reference CSE-20-18-COVID19). All patients admitted to the AP-HP were informed of the possible reuse of their EHRs for research purposes according to the European General Data Protection Regulation and had the right to opt out of participating, in agreement with the Commission Nationale de l'Informatique et des Libertés (regulatory decision DE-2018-155).

## Results

### Population Characteristics

Of 39 different hospitals, 16 (41%) were retained for the study based on the availability of drug exposure information from computerized physician order entries. In these 16 hospitals, we found a total of 8922 eligible patients, of which 3139 (35.18%) were excluded because of insufficient information regarding drug exposure, lab tests, or physiological measurements (see Figure 1). Included and excluded patients were comparable for age (median age 69.2 [IQR 56.7-81.1] vs 70.9 [IQR 55.8-83.8] years) and number of comorbidities (2731/5783 [47.22%] vs 1591/3139 [50.68%] patients with at least 3 comorbidities) but were more often male (3390/5783 [58.62%] vs 1599/3139 [50.94%]); see Table S1 in Multimedia Appendix 1.

A total of 5783 patients were included in the analysis with a median age at admission of 69.2 (IQR 56.7-81.1) years, and 3390 (58.62%) of them were male (Table 1). Patients were admitted from 16 hospitals, with 3 (19%) hospitals representing 2758 (47.69%) of patients. Frequent comorbidities included hypertension (n=2065, 35.71%), chronic kidney disease (n=554, 9.58%), atrial fibrillation or flutter (n=458, 7.92%), dyslipidemia (n=357, 6.17%), and ischemic chronic heart disease (n=356, 6.16%); see Table 1.

**Table 1:** Baseline characteristics of the population (N=5783).

Characteristics		Value
Age at diagnostic (years), median (Q1, Q3)		69.2 (56.7, 81.1)
**Age group at diagnostic (years), n (%)**		
	18-39	322 (5.57)
	40-49	538 (9.30)
	50-59	948 (16.39)
	60-69	1184 (20.47)
	70-79	1241 (21.46)
	80+	1550 (26.80)
Gender (male), n (%)		3390 (58.62)
Deaths, n (%)		933 (16.13)
Follow-up (days), median (Q1, Q3)		8.8 (5.2, 14.9)
28-day Mortality, n (%)		635 (10.98)
**Center, n (%)**		
	GH A Chenevier-H Mondor	965 (16.69)
	Hôpital Saint Antoine	887 (15.34)
	Hôpital Tenon	849 (14.68)
	Other	3082 (53.29)
**Time period, n (%)**		
	February-July 2020	2187 (37.82)
	August-November 2020	1197 (20.70)
	December 2020-June 2021	2399 (41.48)
**Comorbidities, n (%)**		
	Hypertension	2065 (35.71)
	Severe protein energy malnutrition	655 (11.33)
	Chronic kidney disease	554 (9.58)
	Light or moderate protein energy malnutrition	509 (8.80)
	Atrial fibrillation and flutter	458 (7.92)
	Dyslipidemia	357 (6.17)
	Ischemic chronic heart disease	356 (6.16)
	Deficiency in vitamin D	339 (5.86)
	Presence of cardiac and vascular implants and grafts	306 (5.29)
	Hypothyroidism, unspecified	288 (4.98)
**Other parameters, median (Q1, Q3)**		
	BMI	26.5 (23.4, 30.3)
	Pulsations (/min)	89 (78, 102)
	Diastolic arterial pressure (mmHg)	76 (66, 85)
	Systolic arterial pressure (mmHg)	131 (117, 146)
	Respiratory rate (/min)	24 (20, 28)
	Peripheral capillary oxygen saturation (%)	95 (92, 97)
	Body temperature (°C)	37.4 (36.8, 38.2)
	Hemoglobin (g/dL)	13.10 (11.80, 14.30)
	White blood cell count (10^9^/L)	6.38 (4.79, 8.51)
	Creatinine (µmol/L)	80 (65.00, 105.50)
	Blood urea nitrogen (mmol/L)	6.40 (4.60, 9.50)
	CRP^a^ (mg/L)	69.80 (32.50, 122.20)
	Oxygen blood saturation (%)	95 (92.70, 97.00)
	Fibrinogen (g/L)	5.80 (4.85, 6.82)
	Bicarbonate (mmol/L)	25 (22.00, 27.60)

^a^CRP: C-reactive protein.

### Validation With Treatment of References

Without adjustment, dexamethasone was associated with decreased 28-day mortality in patients under 70 years old (OR 0.40, 95% CI 0.26-0.62, *P*<.001) and with increased 28-day mortality in patients over 70 years old (OR 1.40, 95% CI 1.14-1.71, *P*<.001).

**Figure 2:**
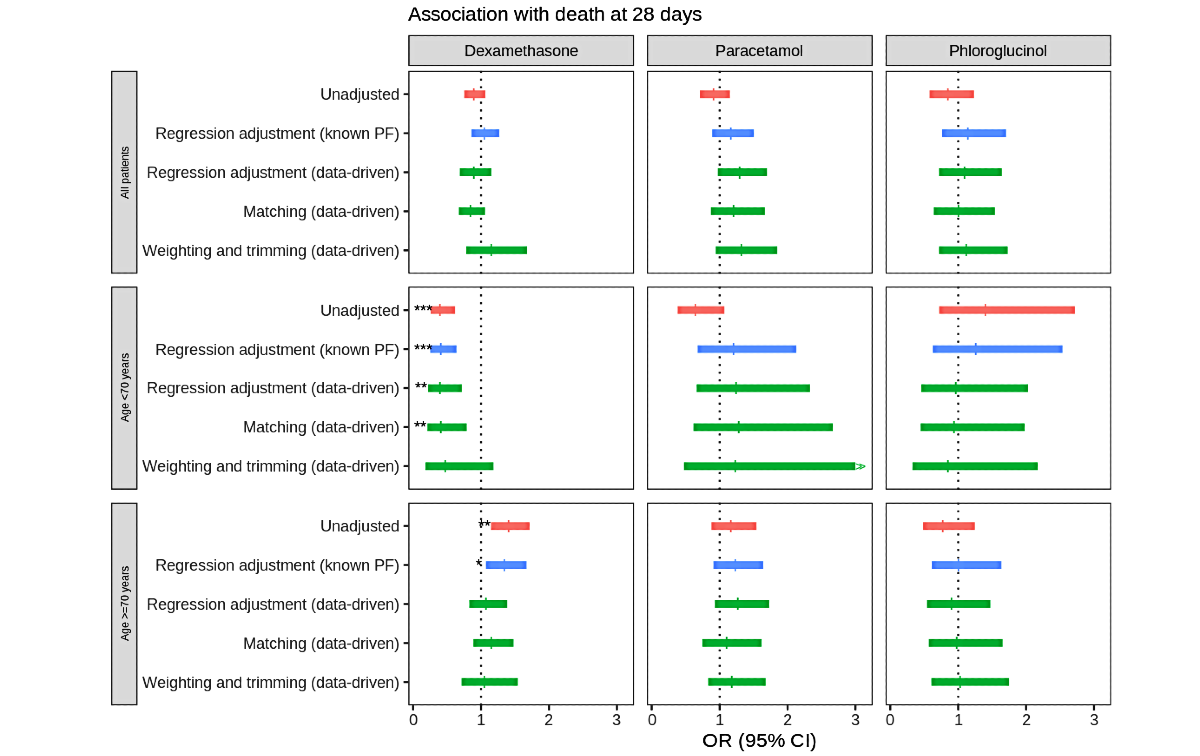
Treatment of references for validating data-driven adjustment set selection. The association between 28-day mortality and early exposure to treatment was measured as the ORs for 3 treatments of reference: (1) dexamethasone with expected beneficial effect on 28-day mortality and (2) treatments with an expected null effect, with high prevalence (paracetamol) or low prevalence (phloroglucinol). We compared 2 pretreatment covariate sets: "known PF" using PFs from the literature (blue) and "data driven" for a model selection procedure based on adaptive LASSO (green) targeting confusion factors. ORs were computed by logistic regression on the overall data set (red), matched or weighted and trimmed subpopulations based on PSs. LASSO: least absolute shrinkage and selection operator; OR: odds ratio; PF: prognostic factor; PS: propensity score. *P<.05; **P<.01; ***P<.001.

Adjusting using the “known PF” and “data driven” adjustments sets yielded close results, except for dexamethasone in patients over 70 years old. In the latter subgroup, only the “data driven” adjustment set yielded no association of dexamethasone with increased 28-day mortality (Figure 2).

On the matched subset for dexamethasone in patients under 70 years old, 3158 (54.61%) of 5783 exposed patients found matches, the association with mortality was strong (OR 0.41, 95% CI 0.21-0.79, *P=*.01), but the FBC was only 35%. On the “weighted and trimmed” subset, the FEP that fell in the EER was only 23.1%, the association with mortality was no longer significant (OR 0.46, 95% CI 0.18-1.18, *P=*.1), but 100% of covariates were balanced.

The fraction of missing information was low and never exceeded 0.2. The FEP was lower than 50% for dexamethasone in all subgroups.

### Pharmacopeia-wide Association With 28-day Mortality

#### Primary Analysis

**Figure 3:**
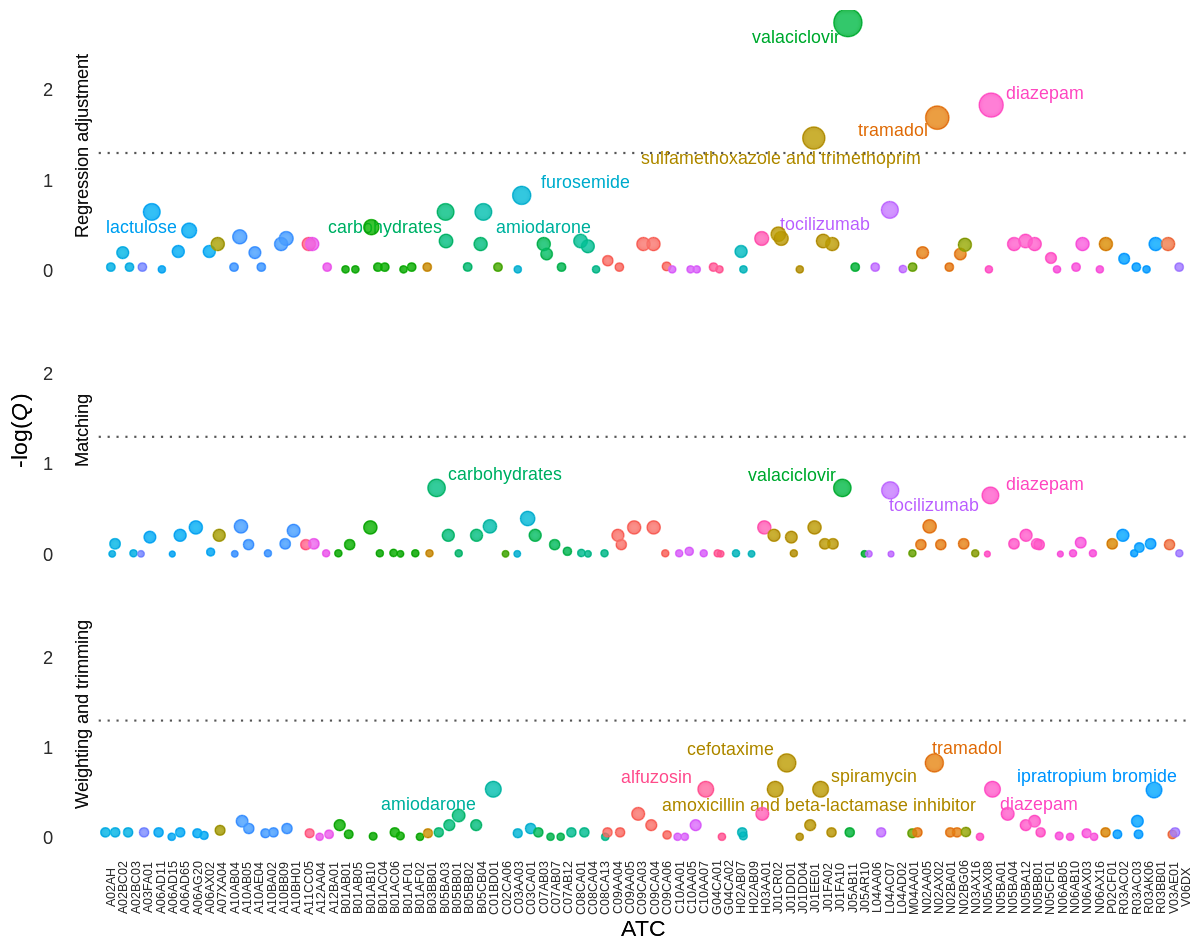
Pharmacopeia-wide association with 28-day mortality. Each dot represents the FDR-corrected *P* value (*Q* value), on a negative log scale (y axis) of a drug (ATC code), on the x axis. An ATC code is attributed if the drug is prescribed in the first 48 hours of COVID-19 admission in conventional wards. The color indicates the pharmacological subgroup (ATC level 2). The top panel reports *Q* values from the primary analysis, using a multivariate logistic regression model, and the dotted line indicates a 5% FDR. The middle and bottom panels report secondary analyses using matching and inverse probability weighting methods, respectively. Dot sizes are inversely proportional to *Q* values. ATC: anatomical therapeutic chemical; FDR: false discovery rate.

**Figure 4:**
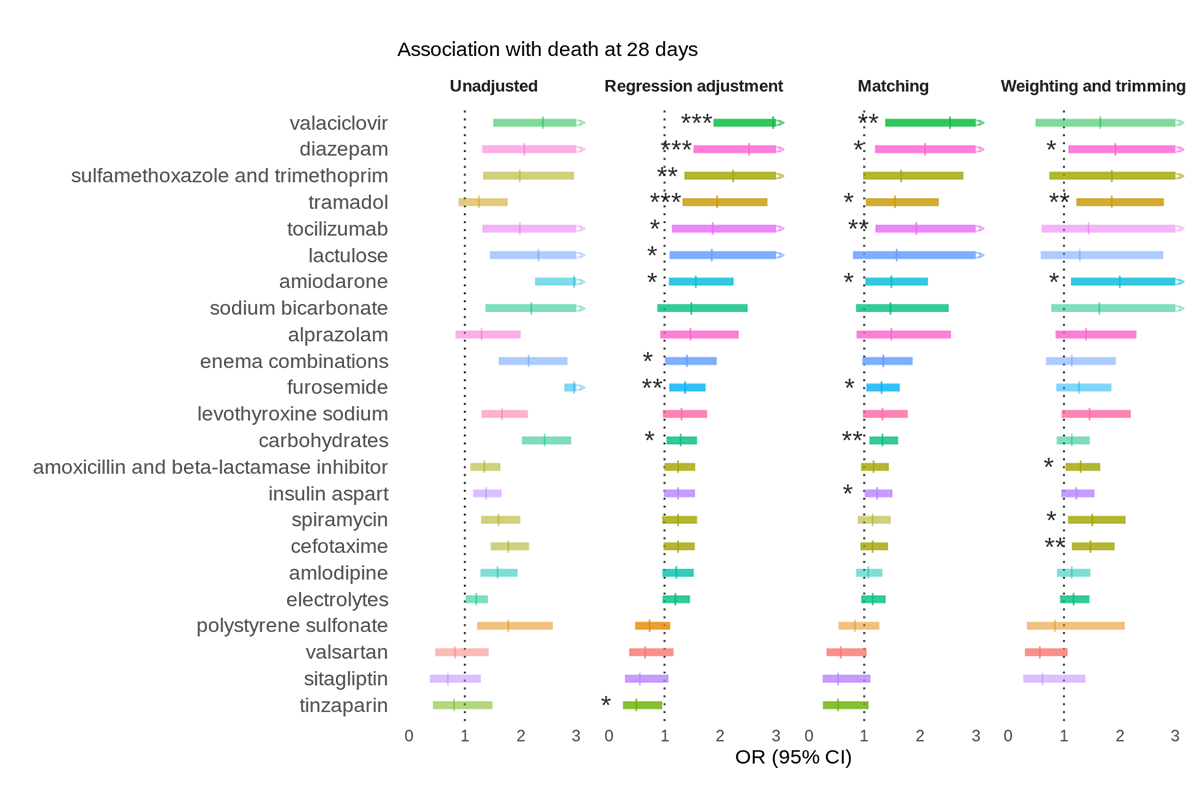
Increased and decreased mortality for the top drugs. Association is reported as the OR between treatment exposition and 28-day mortality in different settings: without adjustment, after adjusting, and on matched and weighted subpopulations based on treatment-specific PSs. p-values are indicated without multiple hypothesis testing correction. Treatments are ordered from top to bottom by decreasing adjusted OR. Drugs at the top tend to be associated with increased mortality, while drugs at the bottom tend to be associated with decreased mortality. Colors correspond to ATC level 2. Only drugs with *P*<.15 are reported. ATC: anatomical therapeutic chemical; OR: odds ratio; PS: propensity score. *P<.05; **P<.01; ***P<.001.

We identified a total of 87 different drugs (ATC level 5codes, eg, B01AF01 rivaroxaban) administered within the first 48 hours and present in at least 100 patient records (Figure 3). Detailed results are given in Figure 4 for drugs with *P*<.15. After correction for multiple hypothesis testing, none were associated with reduced in-hospital mortality, and 4 (5%) remained associated with increased in-hospital mortality on the overall population after adjustment: sulfamethoxazole-trimethoprim, valaciclovir, tramadol, and diazepam (Table 2). Analyses of matched subpopulations found consistent results, with a good fraction of covariate balance (between 98% and 100% of covariates with ASMD<0.1), except for diazepam (89%). Analyses of weighted subpopulations were not consistent and found a small FEP for sulfamethoxazole-trimethoprim and valaciclovir.

**Table 2:** Treatment associated with 28-day mortality after regression adjustment at 5% FDR^a^.

Tests		Treated vs controls (events/exposed), n/n	OR^b^ (95% CI)	*Q* value^c^	FBC^d^ (%)	FEP^e^ (%)	FMI^f^
**Sulfamethoxazole and trimethoprim**							
	Regression adjustment	31/161 vs 604/5622	2.22 (1.36-3.64)	.03	N/A^g^	100	<0.01
	Matching	31/160 vs 63/518	1.65 (0.98-2.78)	N/A	99	99.3	0.03
	Weighting and trimming	8/51 vs 172/1790	1.86 (0.74-4.68)	N/A	91	32.1	0.02
**Valaciclovir**							
	Regression adjustment	24/107 vs 611/5676	3.21 (1.88-5.48)	.002	N/A	100	0.01
	Matching	24/107 vs 41/404	2.54 (1.38-4.67)	N/A	98	99.4	0.08
	Weighting and trimming	6/35 vs 210/2136	1.64 (0.49-5.51)	N/A	71	32.5	0.16
**Tramadol**							
	Regression adjustment	40/302 vs 595/5481	1.94 (1.32-2.85)	.02	N/A	100	<0.01
	Matching	40/301 vs 108/1191	1.55 (1.03-2.34)	N/A	100	99.7	0.08
	Weighting and trimming	31/223 vs 362/4002	1.85 (1.22-2.79)	N/A	100	74	0.02
**Diazepam**							
	Regression adjustment	24/120 vs 611/5663	2.51 (1.52-4.16)	.01	N/A	100	<0.01
	Matching	23/116 vs 47/448	2.09 (1.19-3.66)	N/A	89	96.7	0.06
	Weighting and trimming	15/89 vs 483/4925	1.92 (1.08-3.41)	N/A	100	74.3	0.01

^a^FDR: false discovery rate.

^b^OR: odds ratio

^c^*Q* value: FDR-corrected *P* value.

^d^FBC: fraction of balanced covariates.

^e^FEP: fraction of exposed population.

^f^FMI: fraction of missing information.

^g^N/A: not applicable.

#### Secondary Analysis

We highlight here the results of the weighted and trimmed population where patients were more comparable (Figure 4). Interestingly, 2 angiotensin receptor blockers (ARBs) came up as the top 5 treatments with OR<1, with treatments ordered by *P* values (Table 3). We further explored this hypothesis and found that in weighted and trimmed analysis, ARBs with a high affinity for angiotensin receptor 1 (dissociation constant K_d_≥6: telmisartan, valsartan, losartan) tended to be associated with decreased 28-day mortality compared to ARBs with a lower affinity (K_d_<6: irbesartan, candesartan, olmesartan)—OR 0.56 (95% CI 0.34-0.91).

**Table 3:** Top 5 treatments with OR^a^<1 in the weighted and trimmed population, ordered by *P* value. None were significantly associated with mortality after FDR^b^ correction in the primary analysis.

Treatment	Treated vs controls (events/exposed), n/n	OR (95% CI)	FBC^c^ (%)	FEP^d^ (%)	FMI^e^
Sitagliptin	7/100 vs 426/4023	0.61 (0.27-1.39)	100	71.7	0.01
Valsartan	11/132 vs 562/4567	0.57 (0.30-1.06)	100	87.1	0.01
Irbesartan	32/277 vs 538/4527	0.77 (0.52-1.13)	100	91.4	0.01
Rosuvastatin	12/131 vs 399/3214	0.64 (0.35-1.19)	100	60	0.01
Alfuzosin	6/100 vs 182/1875	0.34 (0.13-0.84)	100	39.3	0.03

^a^OR: odds ratio

^b^FDR: false discovery rate.

^c^FBC: fraction of balanced covariates.

^d^FEP: fraction of exposed population.

^e^FMI: fraction of missing information.

## Discussion

### Principal Findings

We systematically assessed the association of early in-hospital treatments with 28-day mortality in a large, multicenter retrospective case study of 5783 patients with COVID-19, using an innovative PheWAS-like approach. We showed empirical evidence that our fully data-driven pipeline is comparable to or better than a knowledge-based approach to adjust for confounding on 3 drugs of reference. We showed in this practical implementation for COVID-19 how such a pipeline can be used to mine EHR pharmacopoeia and generate pharmacological hypotheses in an exploratory fashion. Indeed, of 87 treatments prescribed in the first 48 hours, 4 (5%) were associated with increased 28-day mortality after adjustment of confounding factors and multiple testing correction, and none were associated with decreased mortality. Among those 4, only diazepam and tramadol had consistent results in secondary analyses more robust to confounding. In addition, secondary analyses suggested that high-affinity ARBs are associated with reduced COVID-19 28-day mortality, suggesting they may be beneficial for patients with COVID-19.

### Validation With Drugs of Reference

We tested our adjustment methods on treatments for which the effect on 28-day mortality is documented (protective effect for dexamethasone) or unlikely to be different from null (absence of an effect for paracetamol and phloroglucinol). Subgroup analysis of the Randomised Evaluation of Covid-19 Therapy (RECOVERY) trial suggests that patients under 70 years old only benefit from dexamethasone, with OR 0.64 (95% CI 0.53−0.78) versus OR 1.03 (95% CI 0.84−1.25) between 70 and 80 years old and OR 0.89 (95% CI 0.75−1.05) above 80 years old [3]. Our automated pipeline finds overall consistent results between the “data driven” and the “known PF” adjustment set for the 3 drugs of reference. The differences observed for the dexamethasone effect on the >70-year age group could be explained by missing or misspecified confounding factors in the “known PF” adjustment set compared to the “data driven” adjustment set (see Table S2 in Multimedia Appendix 1). Overall, these results provide empirical evidence that the automated determination of adjustment sets on these 3 drugs yields valid adjustment sets, sufficient for controlling indication biases.

### Pharmacopeia-wide Association With 28-day Mortality

Interestingly, diazepam, an anxiolytic benzodiazepine, was found to be associated with a detrimental effect on in-hospital mortality in our study. This result might not be COVID-19 specific, as benzodiazepines have shown a dose-response association with mortality in patients with severe chronic obstructive pulmonary disease [46]. We also found that tramadol, a weak opioid, is associated with increased 28-day mortality. Noteworthy, both benzodiazepines and tramadol may have adverse respiratory effects, such as respiratory depression, which, although not specific to COVID-19, could be detrimental in patients with severe COVID-19 pneumonitis [47]. In addition, our automated pipeline allowed us to generate a pharmacological hypothesis consistent with results from a clinical trial. Indeed, an open randomized controlled trial showed that death by day 30 was reduced in patients undergoing telmisartan therapy (control: 16/71 [23%]; telmisartan: 3/70 [4%] participants; *P*=0.002) hospitalized for COVID-19 [48]. However, other studies did not find an association between ARBs and COVID-19 mortality, and further studies are needed to assess this finding and investigate potential mechanisms [49].

### Limits and Strengths

This retrospective study methodology was based on reusing routinely collected clinical data across 16 hospitals of the Greater Paris area. Unexpectedly, from an initial set of 8922 COVID-19 patient records, only 5783 (64.82%) patient records ended up meeting all inclusion criteria. However, this is not intrinsically linked to our method but rather to the relative lack of maturity of the hospitals’ information systems, particularly concerning drug prescription. Indeed, at the beginning of the pandemic, computerized physician order entry was not available in all hospitals and units or not linked to the CDW. Although this study followed most of the guidelines provided by Kohane et al [50], such as a multidisciplinary approach, code transparency, and robustness against variability across hospitals, this result stresses that data completeness in EHRs remains an open question. We can hypothesize that the pandemic will have a boosting effect on the maturation of the information system of hospitals. Regarding confusion adjustment, we could have used more flexible models to fit PSs, such as random forests, and used double robust estimators, which are less sensible to model misspecification [26]. However, we found that the most important factors for accurate measures of treatment association with mortality were the choice of adjustment sets and the use of trimming. Moreover, we decided to restrict to methods that would easily scale to large sets of exposures. Globally, our results are dependent, as in all complex analysis of real-life data, on choices in the preprocessing and modelling of the data. These dependencies can be subtle and lead to changes in amplitude or direction of the measured associations, sometimes framed as “vibration of effect” [51]. Our rationale was to decide these questions based on theoretical grounds (or simulation studies) to leverage treatment of references if not possible (eg, data driven vs knowledge based) and finally to report multiple analyses if uncertainty remains about which method is more relevant (eg, matching or inverse probability weighting).

Large-scale association studies such as this work are known to require a large amount of data to reveal significant associations. Therefore, it may be difficult to obtain sufficient statistical power. To get around this difficulty, it is possible to run the association test using aggregated data to an upper level in the ATC.

Regarding clinical significance, COVID-19 is a biphasic disease, with a viral replication period and then an inflammatory state, and patients may not be hospitalized at the same time of the disease. This may have led to heterogeneity in the condition of the patients and complicated the interpretation of the results. Furthermore, there is a potential risk of selection bias since we dropped 35% of COVID-19 admissions due to data missingness. However, excluded patients were comparable in terms of age and number of comorbidities to the patients included in this study (Table S1 in Multimedia Appendix 1), which is in favor of the generalizability of the obtained results. In addition, we cannot rule out that some confounding factors remain unobserved. Secondary analyses based on EER allowed us to partially address this issue, since the sample size remained large enough in this setting to include a broad amount of potential confounding, and we analyzed a rather homogeneous population by excluding patients admitted to intensive care units (ICUs) in the first 48 hours.

The main strength of our study lies in its external validity: it used data collected across 16 different hospitals of the Greater Paris area and included a large number of patients with COVID-19. These characteristics make it likely to capture the variability of populations and disease management in real-life settings. Similarly, we addressed treatment-specific indication biases in a fully data-driven fashion, which we validated empirically on drugs of reference. This methodology based on a hypothesis-free exploration of COVID-19-related EHRs is easily exportable to other settings. Population trimming based on stabilized PSs allowed us to restrict the analysis to comparable patients, which cannot be done by a simple outcome-oriented regression adjustment. Finally, it allowed us to generate a measure of covariate balancing, which turns out to be a critical diagnostic for studying a large array of drug-outcome associations.

Our systematic hypothesis-free approach constitutes a promising tool that can be rapidly used in the setting of emergent diseases to generate potential drug candidates. Still, these drug candidates need to be further assessed from a pharmacological point of view before being tested in clinical trials. Further developments will include time dependency of treatments, covariates, and outcomes in a more flexible way, not restricted to landmark analysis (28-day mortality) and window-type restriction of exposition. In addition, including information from the natural language processing (NLP) extraction workflow would largely enrich such a pipeline [50,52].

### Conclusion

Our innovative approach proved useful in rapidly generating pharmacological hypotheses in an outbreak setting, without requiring a priori knowledge of the disease. Our systematic analysis of early prescribed treatments from patients hospitalized for COVID-19 showed that diazepam and tramadol are associated with increased 28-day mortality. Whether these drugs could worsen COVID-19 needs to be further assessed.

## Appendix

Multimedia Appendix 1 Supplementary materials.

## Abbreviations

AP-HP: Assistance Publique - Hôpitaux de Paris
ARB: angiotensin receptor blocker
ASMD: absolute standardized mean difference
ATC: anatomical therapeutic chemical
CDW: clinical data warehouse
CRP: C-reactive protein
EDS: Entrepôt de Données de Santé
EER: empirical equipoise region
EHR: electronic health record
FBC: fraction of balanced covariates
FDR: false discovery rate
FEP: fraction of exposed population
FMI: fraction of missing information
ICD-10: International Classification of Diseases, 10th edition
IPW: inverse probability weighting
LASSO: least absolute shrinkage and selection operator
MICE: multiple imputation with chained equations
OR: odds ratio
PF: prognostic factor
PharmWAS: pharmacopeia-wide association study
PheWAS: phenome-wide association study
PS: propensity score
RECOVER: randomised evaluation of covid-19 therapy

## Data Availability

The aggregated results data will be published online, along with the code generating it [53].
