# Correction: Mining Electronic Health Records for Drugs Associated With 28-day Mortality in COVID-19: Pharmacopoeia-wide Association Study (PharmWAS)

**DOI:** 10.2196/38505

**Published:** 2022-04-12

**Authors:** Ivan Lerner, Arnaud Serret-Larmande, Bastien Rance, Nicolas Garcelon, Anita Burgun, Laurent Chouchana, Antoine Neuraz

**Affiliations:** 1 Inserm Centre de Recherche des Cordeliers Sorbonne Université Paris France; 2 Informatique biomédicale Hôpital Necker-Enfants Malades Assistance Publique - Hôpitaux de Paris Paris France; 3 HeKA Team Inria Paris France; 4 Inserm UMR 1163 Data Science Platform Université de Paris, Imagine Institute Paris France; 5 Centre Régional de Pharmacovigilance Service de Pharmacologie, Hôpital Cochin Assistance Publique - Hôpitaux de Paris, Centre - Université de Paris Paris France

In “Mining Electronic Health Records for Drugs Associated With 28-day Mortality in COVID-19: Pharmacopoeia-wide Association Study (PharmWAS)” (JMIR Med Inform 2022;10(3):e35190), the following corrections were made.

1. In the Results section of the abstract, a Q-value was incorrectly written as follows:

Among these, diazepam and tramadol were the only ones not discarded by automated diagnostics, with adjusted odds ratios of 2.51 (95% CI 1.52-4.16, *Q*=.1) and 1.94 (95% CI 1.32-2.85, *Q*=.02), respectively.

This has been corrected to:

Among these, diazepam and tramadol were the only ones not discarded by automated diagnostics, with adjusted odds ratios of 2.51 (95% CI 1.52-4.16, *Q*=.01) and 1.94 (95% CI 1.32-2.85, *Q*=.02), respectively.

2. In the first paragraph of the discussion, the following sentence was incorrectly added as follows:

Indeed, of 87 treatments prescribed in the first 48 hours, 4 (5%) were associated with increased 28-day mortality after adjustment of confounding factors and multiple testing correction, and none were associated with increased mortality.

This has been corrected to:

Indeed, of 87 treatments prescribed in the first 48 hours, 4 (5%) were associated with increased 28-day mortality after adjustment of confounding factors and multiple testing correction, and none were associated with decreased mortality.

The correction will appear in the online version of the paper on the JMIR Publications website on April 12, 2022, together with the publication of this correction notice. Because this was made after submission to PubMed, PubMed Central, and other full-text repositories, the corrected article has also been resubmitted to those repositories.

