# Supplementary material for: Mining Electronic Health Records for Drugs Associated With 28-day Mortality in COVID-19: Pharmacopoeia-wide Association Study (PharmWAS)
Source: JMIR Med Inform. 2022 Mar 30;10(3):e35190. doi: 10.2196/35190 (PMC8970341; doi:10.2196/35190)
Supplement: Multimedia Appendix 1 [file medinform_v10i3e35190_app1.docx]

### **Appendix**

**Table S1: Baseline characteristics by inclusion status**

|  | **Included (N=5783)** | **Excluded (N=3139)** | **Total (N=8922)** |
| --- | --- | --- | --- |
| **Age at diagnostic, years** |  |  |  |
| median [IQR] | 69.18 (56.68, 81.06) | 70.90 (55.79, 83.84) | 69.77 (56.38, 82.16) |
| **Gender, male** | 3390 (58.62%) | 1599 (50.94%) | 4989 (55.92%) |
| **Multiple comorbidities** |  |  |  |
| >=3 | 2731 (47.22%) | 1591 (50.68%) | 4322 (48.44%) |
| **Deaths** | 933 (16.13%) | 599 (19.08%) | 1532 (17.17%) |

**
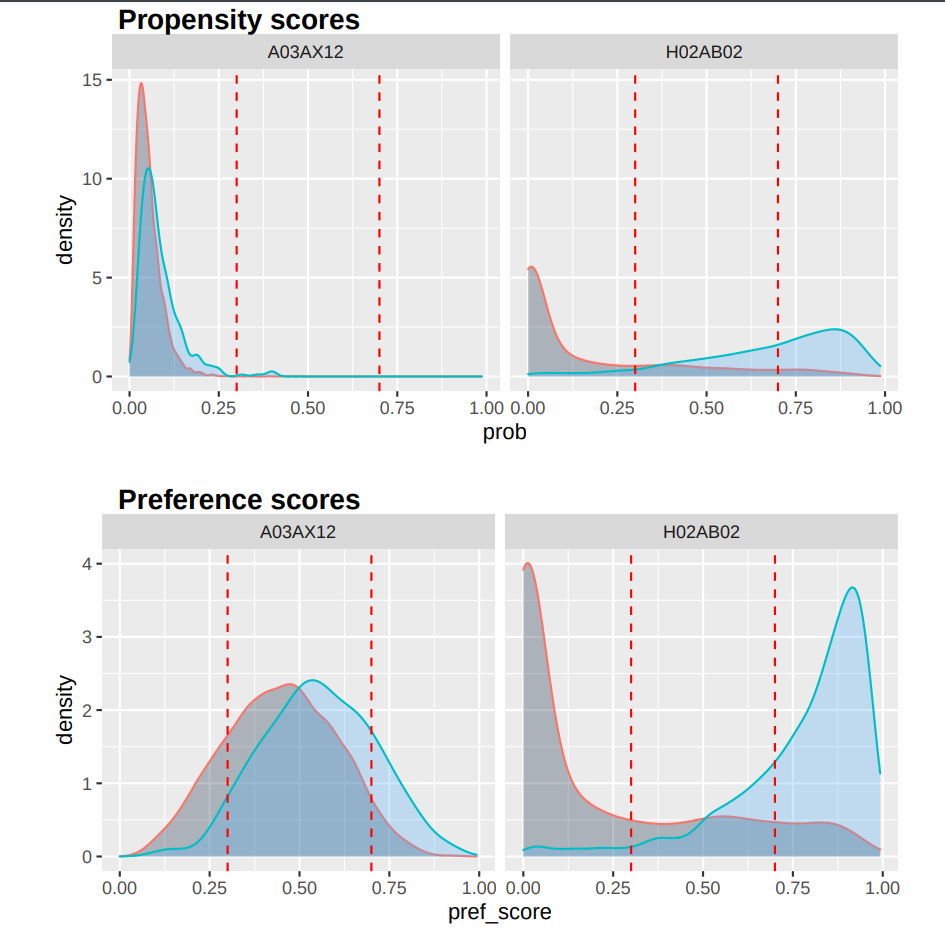
**

**Figure S1. Comparing density plot of propensity score (upper row) and preference score (lower row)**

Left panel (phloroglucinol) and right panel (dexamethasone). Stabilized propensity score or preference scores are propensity scores corrected for prevalence (logit of the preference score equals the logit of the propensity score minus the logit of the drug prevalence. Preference scores allow defining the empirical equipoise region, for patients with preference score between 0.3 and 0.7, where uncertainty among treatment options is strong enough, so that prescribers’ preference drives the prescription instead of only patients’ characteristics.

**Table S2: ‘Data-driven’ versus ‘Knwon PDF’ adjustment sets**

| **Covariate** | **Known PF set** | **Data-driven set** |
| --- | --- | --- |
| **Age** | x | x |
| **Gender** | x |  |
| **Number of comorbidities** | x |  |
| **Platelets** | x |  |
| **Prothrombin ratio** | x |  |
| **Activated partial thromboplastin time (log)** |  | x |
| **Blood urea nitrogen** | x |  |
| **Mean arterial pressure** | x |  |
| **Systolic arterial pressure** | x |  |
| **Brain natriuretic peptide (Q_5)** |  | x |
| **Cancer** |  | x |
| **Creatine kinase (log)** |  | x |
| **Creatine kinase (Q_5)** |  | x |
| **Blood urea nitrogen** | x |  |
| **Creatinin** | x |  |
| **Creatinin (log)** |  | x |
| **Study period (Q_2)** |  | x |
| **Eosinophils** |  | x |
| **Glucose (Q_5)** |  | x |
| **Org. and tissue graft** |  | x |
| **Cardiac insufficiency** |  | x |
| **Missing pH** |  | x |
| **Missing arterial blood saturation** |  | x |
| **Missing D-dimers** |  | x |
| **Missing Lactate** |  | x |
| **Missing pain level** |  | x |
| **Lactate dehydrogenase** |  | x |
| **Lactate (arterial blood) (Q_3)** |  | x |
| **Leukocytes (Q_2)** |  | x |
| **Leukocytes (Q_5)** |  | x |
| **Leukocytes (sq)** |  | x |
| **Alzheimer's disease** |  | x |
| **Liver toxicity** |  | x |
| **Oxygen pressure** |  | x |
| **Oxygen therapy (Q_1)** |  | x |
| **Phosphates (sq)** |  | x |
| **C-reactive protein** | x |  |
| **C-reactive protein (log)** |  | x |
| **Pulse O2 saturation** | x |  |
| **Pulse O2 saturation (Q_3)** |  | x |
| **Malignant neoplasms of male genital organs / urinary tract** |  | x |
| **Endocrine, nutritional and metabolic diseases** |  | x |
| **Ostomy surveillance** |  | x |
| **Symptoms and signs related to mood** |  | x |
| **Body temperature (Q_5)** |  | x |
| **Troponin Ic Hypersensitive (log)** |  | x |
| **Troponin T Ultra sensitive (Q_1)** |  | x |
